# Supplementary material for: A multi-agent reinforcement learning framework for exploring dominant strategies in iterated and evolutionary games
Source: Nat Commun. 2025 Dec 8;17:490. doi: 10.1038/s41467-025-67178-6 (PMC12804933; doi:10.1038/s41467-025-67178-6)
Supplement: Supplementary file 1 — Supplementary Information [file 41467_2025_67178_MOESM1_ESM.pdf]

Supplementary Information for:
A multi-agent reinforcement learning framework for exploring dominant strategies in iterated and evolutionary games

Supplementary Note 1: The detailed formulation of MTBR

The complete formulation of the memory-two bilateral reciprocity (MTBR) strategy is delineated in Supplementary Table 1.

To understand why the table has 20 rows, we need to consider the total number of possible states (N_{state}) in a two-step memory framework. This calculation is more complex than it might initially appear, due to the varying number of states in the initial rounds where full history is not yet available.

Let's break down the calculation:

1. There are 2 individuals, each having $M = 2$ possible actions (cooperate or defect).

2. The maximum memory length is $\ell = 2$.

3. At each interaction step, there are $M \times M = 4$ possible outcomes.

4. However, we need to consider the initial rounds where agents do not have a full history of ℓ steps: - In the first round: 1 state (initial state) - In the second round: $M^2 = 4$ states - From the third round onwards: $M^{2\ell} = 16$ states.

Therefore, the total number of possible states is the sum of all these possibilities:

$N_{\text{state}} = M^2 + M^{2\ell} = 4 + 16 = 20.$

This sum can be generalized and expressed as a geometric series: $N_{\text{state}} = (\frac{M^{2\ell+2}-1}{M^2-1} - 1)$.

For MTBR with $M = 2$ and $\ell = 2$: $N_{\text{state}} = (\frac{2^{2(2)+2}-1}{2^2-1} - 1) = (\frac{63}{3} - 1) = 20$.

This table comprehensively outlines the decision-making processes under a two-step memory framework, presenting each possible state and corresponding strategic responses according to the MTBR's Q-table. Supplementary Table 1 is essential for replicating our findings and understanding the nuanced behavior of agents within the simulated Markov game environments discussed in our study.

Supplementary Note 2: Theoretical analysis

Interaction

We consider the evolutionary dynamics of n strategies, labeled in $1, \cdots, n$, in a well-mixed population of finite and fixed size N. The payoff of these individuals depends on their counterpart's strategies and the payoff matrix

	s_1	s_2	$\cdots$	s_n
s_1	a_{11}	a_{12}	$\cdots$	a_{1n}
s_2	a_{21}	a_{22}	$\cdots$	a_{2n}
$\vdots$	$\vdots$	$\vdots$	$\ddots$	$\vdots$
s_n	a_{n1}	a_{n2}	$\cdots$	a_{nn}

where a_{ij} denotes the payoff received by an i-individual subsequent to the interaction with a j-player. Define the number of i-individuals as X_i and the state vector $\mathbf{X} = (X_1, X_2, \cdots, X_n)$. We know $\sum_{k=1}^{n} X_k = N$. Therefore, we obtain the average payoff of i-individuals

$$\bar{U}_i(\mathbf{X}) = \frac{1}{N-1} \left(a_{i1}X_1 + \cdots + a_{ii}(X_i - 1) + \cdots + a_{in}X_n\right), \tag{1}$$

and the average payoff of all players

$$\bar{U}(\mathbf{X}) = \frac{1}{N} \left(\sum_{k=1}^{n} \bar{U}_i(\mathbf{X}) X_k \right). \tag{2}$$

Strategy updates

After all interactions, a random player i is selected to update his strategy, and another random player j is selected. Player i imitates player j's strategy with probability

$$p_{i \to j} = \frac{1}{1 + \exp\left(\delta\left(\bar{U}_i - \bar{U}_j\right)\right)}. \tag{3}$$

Under weak selection, the probability can be expanded as

$$p_{i\to j} = \frac{1}{2} + \delta\frac{\bar{U}_j - \bar{U}_i}{4} + O(\delta^2). \tag{4}$$

The probability that the number of i-individual increases from X_i to $X_i + 1$ is

$$T_i^+(\mathbf{X}) = \sum_{j\neq i} p_{j\to i}\frac{X_i X_j}{N(N-1)}, \tag{5}$$

whereas probability that the number of i-individual decreases from X_i to $X_i - 1$ is

$$T_i^-(\mathbf{X}) = \sum_{j\neq i} p_{i\to j}\frac{X_i X_j}{N(N-1)}. \tag{6}$$

Under weak selection, we can rewrite the equations as

$$T_i^+(\mathbf{X}) = \sum_{j\neq i}\left(\frac{1}{2} + \delta\frac{\bar{U}_i - \bar{U}_j}{4}\right)\frac{X_i X_j}{N(N-1)}, \tag{7}$$

$$T_i^-(\mathbf{X}) = \sum_{j\neq i}\left(\frac{1}{2} + \delta\frac{\bar{U}_j - \bar{U}_i}{4}\right)\frac{X_i X_j}{N(N-1)}. \tag{8}$$

Evolutionary dynamics

The stochastic evolution process can be formulated in terms of the master equation

$$\begin{aligned} P^{\tau+1}(\mathbf{X}) - P^{\tau}(\mathbf{X}) = &\sum_{i=1}^{n} P^{\tau}(X_1,\cdots,X_i-1,\cdots,X_n)T^+(X_1,\cdots,X_i-1,\cdots,X_n) \\ &+ \sum_{i=1}^{n} P^{\tau}(X_1,\cdots,X_i+1,\cdots,X_n)T^-(X_1,\cdots,X_i+1,\cdots,X_n) \\ &- \sum_{i=1}^{n} P^{\tau}(\mathbf{X})T^+(\mathbf{X}) - \sum_{i=1}^{n} P^{\tau}(\mathbf{X})T^-(\mathbf{X}), \end{aligned} \tag{9}$$

where $P^{\tau}(\mathbf{X})$ is the probability that the system is in state $\mathbf{X}$ at time τ. Introducing the notation $x_i = X_i/N$, $\mathbf{x} = \mathbf{X}/N$, $t = \tau/N$, and the probability density function $\rho(\mathbf{x}, t) = NP^{\tau}(\mathbf{X})$

yields

$$
\begin{aligned}
&\rho\left(\mathbf{x}, t+N^{-1}\right)-\rho(\mathbf{x}, t) \\
=&\sum_{i=1}^{n} \rho\left(x_1, \cdots, x_i-N^{-1}, \cdots, x_n, t\right) T_i^{+}\left(x_1, \cdots, x_i-N^{-1}, \cdots, x_n\right) \\
&+\sum_{i=1}^{n} \rho\left(x_1, \cdots, x_i+N^{-1}, \cdots, x_n, t\right) T_i^{-}\left(x_1, \cdots, x_i+N^{-1}, \cdots, x_n\right) \\
&-\sum_{i=1}^{n} \rho(\mathbf{x}, t) T_i^{-}(\mathbf{x})-\sum_{i=1}^{n} \rho(\mathbf{x}, t) T_i^{+}(\mathbf{x}) .
\end{aligned}
\tag{10}
$$

The probability density function and the transition probability can be expanded in a Taylor series at $(\mathbf{x}, t)$ for large N. Negelecting high order terms in N^{-1}, we get

$$
\begin{aligned}
&\frac{1}{N} \frac{\partial}{\partial t} \rho(\mathbf{x}, t) \\
=&\sum_{i=1}^{n}\left(\rho(\mathbf{x}, t)-\frac{1}{N} \frac{\partial}{\partial x_i} \rho(\mathbf{x}, t)+\frac{1}{2 N^2} \frac{\partial^2}{\partial x_i^2} \rho(\mathbf{x}, t)\right)\left(T_i^{+}(\mathbf{x})-\frac{1}{N} \frac{\partial}{\partial x_i} T_i^{+}(\mathbf{x})+\frac{1}{2 N^2} \frac{\partial^2}{\partial x_i^2} T_i^{+}(\mathbf{x})\right) \\
&+\sum_{i=1}^{n}\left(\rho(\mathbf{x}, t)+\frac{1}{N} \frac{\partial}{\partial x_i} \rho(\mathbf{x}, t)+\frac{1}{2 N^2} \frac{\partial^2}{\partial x_i^2} \rho(\mathbf{x}, t)\right)\left(T_i^{-}(\mathbf{x})+\frac{1}{N} \frac{\partial}{\partial x_i} T_i^{-}(\mathbf{x})+\frac{1}{2 N^2} \frac{\partial^2}{\partial x_i^2} T_i^{-}(\mathbf{x})\right) \\
&-\sum_{i=1}^{n} \rho(\mathbf{x}, t) T_i^{-}(\mathbf{x})-\sum_{i=1}^{n} \rho(\mathbf{x}, t) T_i^{+}(\mathbf{x}) \\
=&-\frac{1}{N} \sum_{i=1}^{n} \frac{\partial}{\partial x_i}\left(\phi_i(\mathbf{x}) \rho(\mathbf{x}, t)\right)+\frac{1}{2 N} \sum_{i=1}^{n} \frac{\partial^2}{\partial x_i^2}\left(\psi_i^2(\mathbf{x}) \rho(\mathbf{x}, t)\right) .
\end{aligned}
\tag{11}
$$

Finally, we obtain

$$
\frac{\partial}{\partial t} \rho(\mathbf{x}, t)=-\sum_{i=1}^{n} \frac{\partial}{\partial x_i}\left(\phi_i(\mathbf{x}) \rho(\mathbf{x}, t)\right)+\sum_{i=1}^{n} \frac{\partial^2}{\partial x_i^2}\left(\psi_i^2(\mathbf{x}) \rho(\mathbf{x}, t)\right), \tag{12}
$$

where

$$
\phi_i(\mathbf{x})=T_i^{+}(\mathbf{x})-T_i^{-}(\mathbf{x})=\frac{\delta N}{2(N-1)} x_i\left(\bar{U}_i-\bar{U}\right), \tag{13a}
$$

$$
\psi_i(\mathbf{x})=\sqrt{\left(T_i^{+}(\mathbf{x})+T_i^{-}(\mathbf{x})\right) / N}=\sqrt{x_i\left(1-x_i\right) /(N-1)}. \tag{13b}
$$

The partial differential equation has the form of a Fokker-Planck equation. Meanwhile, we can derive the corresponding Langevin equation

$$\dot{x}_i = \phi_i(\mathbf{x}) + \psi_i(\mathbf{x})\xi, \tag{14}$$

where ξ is the Gaussian noise.

Equilibrium point

The equilibrium points are determined by the first term of Eq. 14, while the second term affects the stability. To find all equilibrium points, we can omit the second term and let $\dot{x}_i = 0$ for all $i \in \{1, 2, \cdots, n\}$, from which we know there are n boundary equilibrium points $(1, 0, \cdots 0), (0, 1, \cdots, 0), \cdots, (0, 0, \cdots, 1)$. In scenarios where interior equilibrium points exist, we discuss the following cases.

All strategies coexist

In this case, the following equations must hold

$$\bar{U}_1(\mathbf{x}) = \bar{U}_2(\mathbf{x}) = \cdots = \bar{U}_n(\mathbf{x}), \tag{15a}$$

$$x_1 + x_2 + \cdots + x_n = 1. \tag{15b}$$

Moreover, we get a system of linear equations

$$\begin{cases} x_1(a_{11} - a_{21}) + x_2(a_{12} - a_{22}) + \cdots + x_n(a_{1n} - a_{2n}) = \dfrac{a_{11} - a_{22}}{N}, \\ x_1(a_{11} - a_{31}) + x_2(a_{12} - a_{32}) + \cdots + x_n(a_{1n} - a_{3n}) = \dfrac{a_{11} - a_{33}}{N}, \\ \qquad \vdots \qquad\qquad\qquad \vdots \qquad\qquad\qquad \vdots \\ x_1(a_{11} - a_{n1}) + x_2(a_{12} - a_{n2}) + \cdots + x_n(a_{1n} - a_{nn}) = \dfrac{a_{11} - a_{nn}}{N}, \\ x_1 + x_2 + \cdots + x_n = 1, \end{cases} \tag{16}$$

where $0 < x_i < 1, \forall i \in \{1, 2, \cdots, n\}$. Rewrite these equations in the matrix form

$$\mathbf{A}\mathbf{x} = \mathbf{b}, \tag{17}$$

where

$$\mathbf{A} = \begin{pmatrix} a_{11}-a_{21} & a_{12}-a_{22} & \cdots & a_{1n}-a_{2n} \\ a_{11}-a_{31} & a_{12}-a_{32} & \cdots & a_{1n}-a_{3n} \\ \vdots & \vdots & \ddots & \vdots \\ a_{11}-a_{n1} & a_{12}-a_{n2} & \cdots & a_{1n}-a_{nn} \\ 1 & 1 & \cdots & 1 \end{pmatrix}, \tag{18}$$

$$\mathbf{b} = \begin{pmatrix} (a_{11}-a_{22})/N \\ (a_{11}-a_{33})/N \\ \vdots \\ (a_{11}-a_{nn})/N \\ 1 \end{pmatrix}. \tag{19}$$

Therefore, if $|\mathbf{A}| \neq 0$ holds, there may exist an interior equilibrium point. According to the Cramer's rule, we obtain

$$\begin{aligned} x_1 &= \frac{|\mathbf{A}_1|}{|\mathbf{A}|}, \\ x_2 &= \frac{|\mathbf{A}_2|}{|\mathbf{A}|}, \\ &\vdots \\ x_n &= \frac{|\mathbf{A}_n|}{|\mathbf{A}|}, \end{aligned} \tag{20}$$

where $\mathbf{A}_i$ is the matrix formed by replacing the i_{th} column of $\mathbf{A}$ by the column vector $\mathbf{b}$.

m strategies coexist

Denote the fraction of remaining strategies as $x_{i_1}, x_{i_2}, \cdots, x_{i_m}$ and the fraction of the extinct strategies $x_{j_1}, x_{j_2}, \cdots, x_{j_{n-m}}$. Let $\mathcal{I} := \{i_1, i_2, \cdots, i_m\}$ and $\mathcal{J} := \{j_1, j_2, \cdots, j_m\}$. In this case, the following equations must hold

$$\bar{U}_{i_1}(\mathbf{x}) = \bar{U}_{i_2}(\mathbf{x}) = \cdots = \bar{U}_{i_m}(\mathbf{x}), \tag{21a}$$

$$x_{i_1} + x_{i_2} + \cdots + x_{i_m} = 1, \tag{21b}$$

$$x_{j_1} = x_{j_2} = \cdots = x_{j_{n-m}} = 0. \tag{21c}$$

Similarly, we get a system of linear equations

$$\begin{cases} x_{i_1}(a_{i_1 i_1} - a_{i_2 i_1}) + x_{i_2}(a_{i_1 i_2} - a_{i_2 i_2}) + \cdots + x_{i_m}(a_{i_1 i_m} - a_{i_2 i_m}) = \dfrac{a_{i_1 i_1} - a_{i_2 i_2}}{N}, \\ x_{i_1}(a_{i_1 i_1} - a_{i_3 i_1}) + x_{i_3}(a_{i_1 i_2} - a_{i_3 i_2}) + \cdots + x_{i_m}(a_{i_1 i_m} - a_{i_3 i_m}) = \dfrac{a_{i_1 i_1} - a_{i_3 i_3}}{N}, \\ \qquad \vdots \qquad\qquad \vdots \qquad\qquad \vdots \\ x_{i_1}(a_{i_1 i_1} - a_{i_m i_1}) + x_{i_2}(a_{i_1 i_2} - a_{i_m i_2}) + \cdots + x_{i_m}(a_{i_1 i_m} - a_{i_m i_m}) = \dfrac{a_{i_1 i_1} - a_{i_m i_m}}{N}, \\ x_{i_1} + x_{i_2} + \cdots + x_{i_m} = 1, \end{cases} \tag{22}$$

where $0 < x_{i_k} < 1, \forall i_k \in \mathcal{I}$. These equations can also be rewritten in the matrix form

$$\mathbf{A}_{\mathcal{I}} \mathbf{x}_{\mathcal{I}} = \mathbf{b}_{\mathcal{I}}, \tag{23}$$

where

$$\mathbf{A}_{\mathcal{I}} = \begin{pmatrix} a_{i_1 i_1} - a_{i_2 i_1} & a_{i_1 i_2} - a_{i_2 i_2} & \cdots & a_{i_1 i_m} - a_{i_2 i_m} \\ a_{i_1 i_1} - a_{i_3 i_1} & a_{i_1 i_2} - a_{i_3 i_2} & \cdots & a_{i_1 i_m} - a_{i_3 i_m} \\ \vdots & \vdots & \ddots & \vdots \\ a_{i_1 i_1} - a_{i_m i_1} & a_{i_1 i_2} - a_{i_m i_2} & \cdots & a_{i_1 i_m} - a_{i_m i_m} \\ 1 & 1 & \cdots & 1 \end{pmatrix}, \tag{24}$$

$$\mathbf{b}_{\mathcal{I}} = \begin{pmatrix} (a_{i_1 i_1} - a_{i_2 i_2})/N \\ (a_{i_1 i_1} - a_{i_3 i_3})/N \\ \vdots \\ (a_{i_1 i_1} - a_{i_m i_m})/N \\ 1 \end{pmatrix}. \tag{25}$$

Therefore, if $|\mathbf{A}_{\mathcal{I}}| \neq 0$ holds, there may exist an interior equilibrium point. According to Cramer's rule, we obtain the following equations

$$\begin{aligned} x_{i_1} &= \frac{|\mathbf{A}_{\mathcal{I}1}|}{|\mathbf{A}_{\mathcal{I}}|}, \\ x_{i_2} &= \frac{|\mathbf{A}_{\mathcal{I}2}|}{|\mathbf{A}_{\mathcal{I}}|}, \\ &\vdots \\ x_{i_m} &= \frac{|\mathbf{A}_{\mathcal{I}m}|}{|\mathbf{A}_{\mathcal{I}}|}, \end{aligned} \tag{26}$$

where $\mathbf{A}_{\mathcal{I}k}$ is the matrix formed by replacing the k_{th} column of $\mathbf{A}_{\mathcal{I}}$ by the column vector $\mathbf{b}_{\mathcal{I}}$.

Note that we may get some equilibrium points where there exist negative elements. Therefore, we should check the results after solving the equations.

Stability

Define $f_i(\mathbf{x}) = x_i(\bar{U}_i - \bar{U})$. Obviously, $\dot{f}_i$ and $\ddot{x}_i$ have the same sign. So we can use $f_i(\mathbf{x})$ to analyze the stability of the system. We rewrite Eq. 1 and Eq. 2 in the following form

$$\bar{U}_i = \frac{N}{N-1}\sum_{j=1}^{n} a_{ij}\left(x_j - \frac{1}{N}\delta_{ij}\right), \tag{27}$$

$$\bar{U} = \frac{N}{N-1}\sum_{k=1}^{n}\sum_{j=1}^{n} a_{ij}\left(x_j - \frac{1}{N}\delta_{ij}\right)x_k, \tag{28}$$

from which we get

$$\begin{aligned} \frac{\partial\left(\bar{U}_i - \bar{U}\right)}{\partial x_m} &= \frac{N}{N-1}a_{im} - \frac{N}{N-1}\sum_{j\neq m} a_{mj}x_j - \frac{N}{N-1}\sum_{k\neq m} a_{km}x_k - \frac{N}{N-1}a_{mm}\left(2x_m - \frac{1}{N}\right) \\ &= \frac{N}{N-1}a_{im} - \frac{N}{N-1}\sum_{k=1}^{n}(a_{km} + a_{mk})x_k + \frac{1}{N-1}a_{mm}. \end{aligned} \tag{29}$$

Thus, we obtain

$$\frac{\partial f_i(\mathbf{x})}{\partial x_m} = \frac{N}{N-1}\left(a_{im} + \frac{1}{N}a_{mm} - \sum_{k=1}^{n} x_k(a_{km} + a_{mk})\right)x_i + \delta_{im}\left(\bar{U}_i - \bar{U}\right), \tag{30}$$

where $\delta_{im} = 1$ if $i = m$ otherwise $\delta_{im} = 0$. Define the Jacobian matrix

$$\mathbf{J}(\mathbf{x}) = \begin{pmatrix} \frac{\partial f_1(\mathbf{x})}{\partial x_1} & \frac{\partial f_1(\mathbf{x})}{\partial x_2} & \cdots & \frac{\partial f_1(\mathbf{x})}{\partial x_n} \\ \frac{\partial f_2(\mathbf{x})}{\partial x_1} & \frac{\partial f_2(\mathbf{x})}{\partial x_2} & \cdots & \frac{\partial f_2(\mathbf{x})}{\partial x_n} \\ \vdots & \vdots & \ddots & \vdots \\ \frac{\partial f_n(\mathbf{x})}{\partial x_1} & \frac{\partial f_n(\mathbf{x})}{\partial x_2} & \cdots & \frac{\partial f_n(\mathbf{x})}{\partial x_n} \end{pmatrix}. \tag{31}$$

For a given equilibrium point $\mathbf{x}^*$, it is stable if and only if all elements of $\mathbf{J}(\mathbf{x})$ are negative.

Supplementary Note 3: When MTBR is a partner strategy

We consider the infinitely repeated Prisoner's Dilemma (IPD) between two players, Player 1 and Player 2. In each round, both players simultaneously choose either to cooperate (C) or to defect (D), and this interaction continues for infinitely many rounds. The payoff matrix is given by

$$\begin{array}{c|cc} & C & D \\ \hline C & R & S \\ D & T & P \end{array} \tag{32}$$

Here, R denotes the reward for mutual cooperation, T the temptation payoff for unilateral defection, S the sucker's payoff, and P the punishment for mutual defection. Throughout, we restrict attention to strategies with finite memory.

Our goal is to identify strategies that can sustain mutual cooperation as a Nash equilibrium. Such strategies have been termed *partner strategies* by Hilbe (Nature Human Behaviour, 2018, 2(7): 469–477). Specifically, in the repeated Prisoner's Dilemma with $T + S < 2R$, a player (say, Alice) is said to use a partner strategy if the following two conditions are satisfied:

1. If Bob adopts the same strategy as Alice, both players receive the mutual cooperation payoff: $\pi_A = \pi_B = R$.

2. By applying a different strategy, Bob can get at most R, in which case Alice gets the same payoff. Otherwise, Bob gets a payoff lower than R.

In what follows, we analyze the conditions under which the MTBR strategy satisfies these criteria and thus qualifies as a partner strategy.

Before presenting the proof, we introduce two key preliminaries that underpin our analysis.

1. Memory compression. Press and Dyson (PNAS, 2012, 109(26), 10409–10413) showed that when two players have different memory lengths, the player with the longer memory can achieve the same payoff by using a shorter-memory strategy that ignores information unavailable to the opponent. This result allows us to simplify asymmetric-memory interactions into equivalent interactions where both players have the same memory length. Therefore, to evaluate how MTBR performs against arbitrary strategies, it suffices to consider its performance against opponents with memory-2 strategies.

2. Pure strategy best response. When both players use memory-2 strategies, the best response to any fixed strategy is always achieved by a pure strategy. This extends the reasoning originally developed by Press and Dyson for memory-1 strategies. Specifically, the expected payoff can be written as a linear-fractional function of the opponent's strategy — meaning both the numerator and denominator are linear in the opponent's strategy variables. Since the strategy space is a compact, convex hypercube $[0,1]^{16}$, the maximum is always attained at one of its corners, i.e., a pure strategy.

Sketch of Proof. (**Pure strategy best response**) Let $\mathbf{p}_1$ and $\mathbf{p}_2$ denote the strategies of Player 1 and Player 2, respectively. The state transition matrix M for the joint Markov process induced by these strategies can be expressed in terms of these two vectors.

After subtracting the identity matrix, the matrix $M' = M - I$ is singular. Following the method of Press and Dyson (PNAS, 2012, 109(26), 10409–10413), the expected payoffs of both players can be written in the form:

$$\pi_1 = \frac{D(\mathbf{p}', \mathbf{q}', \mathbf{s}_1')}{D(\mathbf{p}', \mathbf{q}', \mathbf{1})}, \qquad \pi_2 = \frac{D(\mathbf{p}', \mathbf{q}', \mathbf{s}_2')}{D(\mathbf{p}', \mathbf{q}', \mathbf{1})}, \tag{33}$$

where $D(\mathbf{p}', \mathbf{q}', \mathbf{s})$ denotes the determinant of the modified matrix obtained by replacing the final column of M''s adjoint matrix with the vector $\mathbf{s}$; $\mathbf{p}'$ and $\mathbf{q}'$ are the rearranged strategies of Player 1 and Player 2; $\mathbf{s}_1'$ and $\mathbf{s}_2'$ are the corresponding payoff vectors for Player 1 and Player 2, and $\mathbf{1}$ is the all-ones vector.

By properties of the determinant, both the numerator and denominator of the expected payoff expression are linear in the elements of $\mathbf{q}$. Therefore, each player's expected payoff is a monotonic function with respect to the opponent's strategy.

Consequently, given a fixed strategy of one player, the best response of the other must lie at a vertex of the strategy space, i.e., it must be a pure strategy. □

With these two preliminaries in place, we only need to show that MTBR satisfies the partner conditions when interacting with any of the 2^{16} possible pure memory-2 strategies. We represent the interaction history of player 1 and player 2 both adopting memory-2 strategy at time t as

$$\left(\left(a_1^{t-2}, a_2^{t-2}\right), \left(a_1^{t-1}, a_2^{t-1}\right)\right), \tag{34}$$

where $a = 1$ indicates cooperation and $a = 0$ indicates defection. This interaction state is then

mapped to a scalar index using the formula

$$i = 15 - \left(2^0 \cdot a_2^{t-1} + 2^1 \cdot a_1^{t-1} + 2^2 \cdot a_2^{t-2} + 2^3 \cdot a_1^{t-2}\right). \tag{35}$$

For example, if both players cooperated in each of the last two rounds, the state index is

$$i = 15 - (2^0 + 2^1 + 2^2 + 2^3) = 15 - 15 = 0. \tag{36}$$

The complete mapping between the interaction state and the corresponding index is provided in Supplementary Table 2.

A memory-2 strategy is then defined as a 16-dimensional vector

$$\mathbf{p} = (p_0, p_1, p_2, \ldots, p_{15}), \tag{37}$$

where p_i denotes the probability of cooperating when the current interaction state is i. In particular, the memory-2 strategy MTBR, when adopted by Player 1, is given by

$$\mathbf{p}_{\text{MTBR}} = (1,0,1,0,1,0,1,0,1,0,1,0,1,0,1,1). \tag{38}$$

We denote $v_i(t)$ as the probability that the joint actions of the two players over the last two rounds correspond to state i. We can compute the stationary distribution $\mathbf{v}$ induced by the interaction between MTBR (as Player 1) and a given memory-2 strategy (as Player 2). The stationary distribution satisfies

$$\begin{aligned} \mathbf{v}^T M &= \mathbf{v}^T, \\ \mathbf{v}^T \mathbf{1} &= 1, \end{aligned} \tag{39}$$

where M is the state transition matrix over the 16 interaction history.

Given $\mathbf{v}$, the expected payoff for MTBR is computed as

$$\pi_1 = \mathbf{v}^T \cdot \mathbf{g}_1, \tag{40}$$

and the expected payoff for the opponent is

$$\pi_2 = \mathbf{v}^T \cdot \mathbf{g}_2, \tag{41}$$

where the payoff vectors for Player 1 and Player 2 are defined as:

$$\mathbf{g}_1 = (R, S, T, P, R, S, T, P, R, S, T, P, R, S, T, P)\,, \tag{42}$$

$$\mathbf{g}_2 = (R, T, S, P, R, T, S, P, R, T, S, P, R, T, S, P)\,. \tag{43}$$

To determine whether MTBR is a partner strategy, we check that the second condition holds—i.e., the opponent's expected payoff does not exceed the mutual cooperation payoff R. Through exhaustive evaluation over all 2^{16} memory-2 pure strategies, we identify the set of inequalities under which this condition is satisfied:

$$\begin{aligned}
&\frac{2P}{3} + \frac{T}{3} < R,\\
&\frac{2P}{5} + \frac{2R}{5} + \frac{T}{5} < R,\\
&\frac{2P}{5} + \frac{S}{5} + \frac{2T}{5} < R,\\
&\frac{2P}{7} + \frac{2R}{7} + \frac{S}{7} + \frac{2T}{7} < R,\\
&\frac{P}{2} + \frac{R}{4} + \frac{T}{4} < R,\\
&\frac{P}{3} + \frac{R}{6} + \frac{S}{6} + \frac{T}{3} < R,\\
&\frac{P}{3} + \frac{S}{3} + \frac{T}{3} < R,\\
&\frac{P}{4} + \frac{R}{4} + \frac{S}{4} + \frac{T}{4} < R,\\
&\frac{P}{5} + \frac{2R}{5} + \frac{S}{5} + \frac{T}{5} < R,\\
&\frac{P}{6} + \frac{R}{6} + \frac{S}{3} + \frac{T}{3} < R,\\
&\frac{P}{7} + \frac{2R}{7} + \frac{2S}{7} + \frac{2T}{7} < R,\\
&\frac{R}{2} + \frac{S}{4} + \frac{T}{4} < R,\\
&\frac{R}{3} + \frac{S}{3} + \frac{T}{3} < R,\\
&\frac{S}{2} + \frac{T}{2} < R.
\end{aligned} \tag{44}$$

These inequalities provide sufficient conditions under which MTBR qualifies as a partner strategy in the IPD.

By reducing the above set of inequalities, we obtain the following compact form:

$$\begin{aligned} &S < R, \\ &T < 2R - S, \\ &P < \frac{1}{2}(3R - T). \end{aligned} \tag{45}$$

In other words, if these three conditions are satisfied, then MTBR guarantees mutual cooperation when facing itself, and simultaneously prevents any opponent from gaining a higher payoff than R while make both players get R when mutual cooperation—thereby fulfilling the criteria for a partner strategy in the IPD.

Furthermore, under the payoff structure of the donation game, where $R = b - c$, $S = -c$, $T = b$, and $P = 0$, these conditions simplify to a threshold condition on the benefit-to-cost ratio:

$$\frac{b}{c} > \left(\frac{b}{c}\right)^* = 1.5. \tag{46}$$

This condition is satisfied in most practical settings, as the requirement $b > c$ is already necessary for the interaction to constitute a Prisoner's Dilemma.

The stationary distributions and the full implementation code used to derive these results are available on GitHub.

Two Steps Ago (Opponent)	Two Steps Ago (Self)	One Step Ago (Opponent)	One Step Ago (Self)	Strategy Choice
Cooperate	Cooperate	Cooperate	Cooperate	Cooperate
Cooperate	Cooperate	Cooperate	Defect	Cooperate
Cooperate	Cooperate	Defect	Cooperate	Defect
Cooperate	Cooperate	Defect	Defect	Defect
Cooperate	Defect	Cooperate	Cooperate	Cooperate
Cooperate	Defect	Cooperate	Defect	Cooperate
Cooperate	Defect	Defect	Cooperate	Defect
Cooperate	Defect	Defect	Defect	Defect
Defect	Cooperate	Cooperate	Cooperate	Cooperate
Defect	Cooperate	Cooperate	Defect	Cooperate
Defect	Cooperate	Defect	Cooperate	Defect
Defect	Cooperate	Defect	Defect	Defect
Defect	Defect	Cooperate	Cooperate	Cooperate
Defect	Defect	Cooperate	Defect	Cooperate
Defect	Defect	Defect	Cooperate	Defect
Defect	Defect	Defect	Defect	Cooperate
-	-	Cooperate	Cooperate	Cooperate
-	-	Cooperate	Defect	Cooperate
-	-	Defect	Cooperate	Cooperate
-	-	Defect	Defect	Defect

Supplementary Table 1: The detailed formulation of MTBR. This table presents the complete decision-making logic of the MTBR strategy based on the last two interactions. Each row represents a unique state, with the first 16 rows showing all possible combinations of full two-step memory, and the last 4 rows representing states with incomplete memory (initial rounds). The ‘Strategy Choice’ column indicates the MTBR agent’s action (Cooperate or Defect) for each state. This shows how MTBR considers both players’ actions over two steps to make decisions, illustrating its sophisticated approach to reciprocity and cooperation in iterated games.

a_1^{t-2}	a_2^{t-2}	a_1^{t-1}	a_2^{t-1}	Index
1	1	1	1	0
1	1	1	0	1
1	1	0	1	2
1	1	0	0	3
1	0	1	1	4
1	0	1	0	5
1	0	0	1	6
1	0	0	0	7
0	1	1	1	8
0	1	1	0	9
0	1	0	1	10
0	1	0	0	11
0	0	1	1	12
0	0	1	0	13
0	0	0	1	14
0	0	0	0	15

Supplementary Table 2: Relationship between interaction state and index.

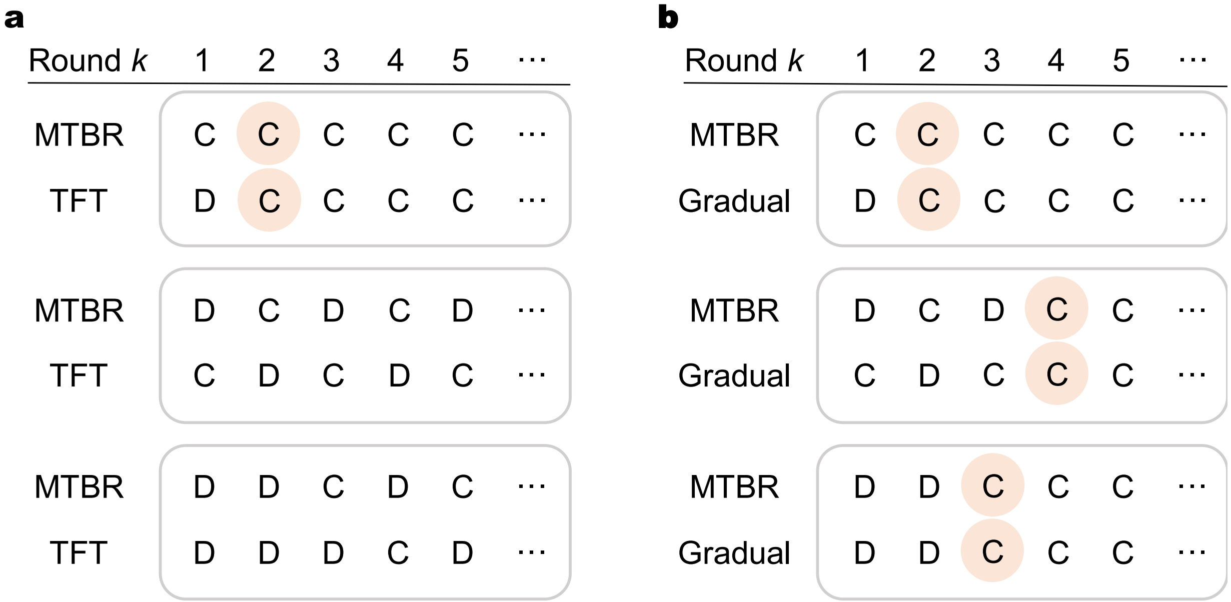

Supplementary Figure 1: Interactions between MTBR, TFT, and GradualTFT in the repeated Prisoner's Dilemma. **a**, Interactions between MTBR and TFT. When MTBR initiates cooperation and TFT defects, MTBR reciprocates cooperatively, leading to mutual cooperation. When MTBR starts with defection and TFT cooperates, they enter a "cooperate-defect" cycle. If both defect initially, MTBR's subsequent cooperation improves the situation to a "cooperate-defect" cycle. This demonstrates MTBR's effectiveness in fostering cooperation when paired with TFT. **b**, Interactions between MTBR and GradualTFT. When MTBR cooperates first and GradualTFT defects, they immediately achieve mutual cooperation. If MTBR defects first while GradualTFT cooperates, mutual cooperation is reached after the third round. When both initially defect, they achieve mutual cooperation after two rounds of goodwill gestures. Yellow circles indicate individuals ceasing defection and entering mutual cooperation. Comparison with panel **a** shows that MTBR and GradualTFT, as cooperative strategies, achieve better mutual cooperation, gaining an advantage in evolutionary games. This figure extends the analysis presented in Fig. 1, providing deeper insights into MTBR's interactions with other strategies in the repeated Prisoner's Dilemma.

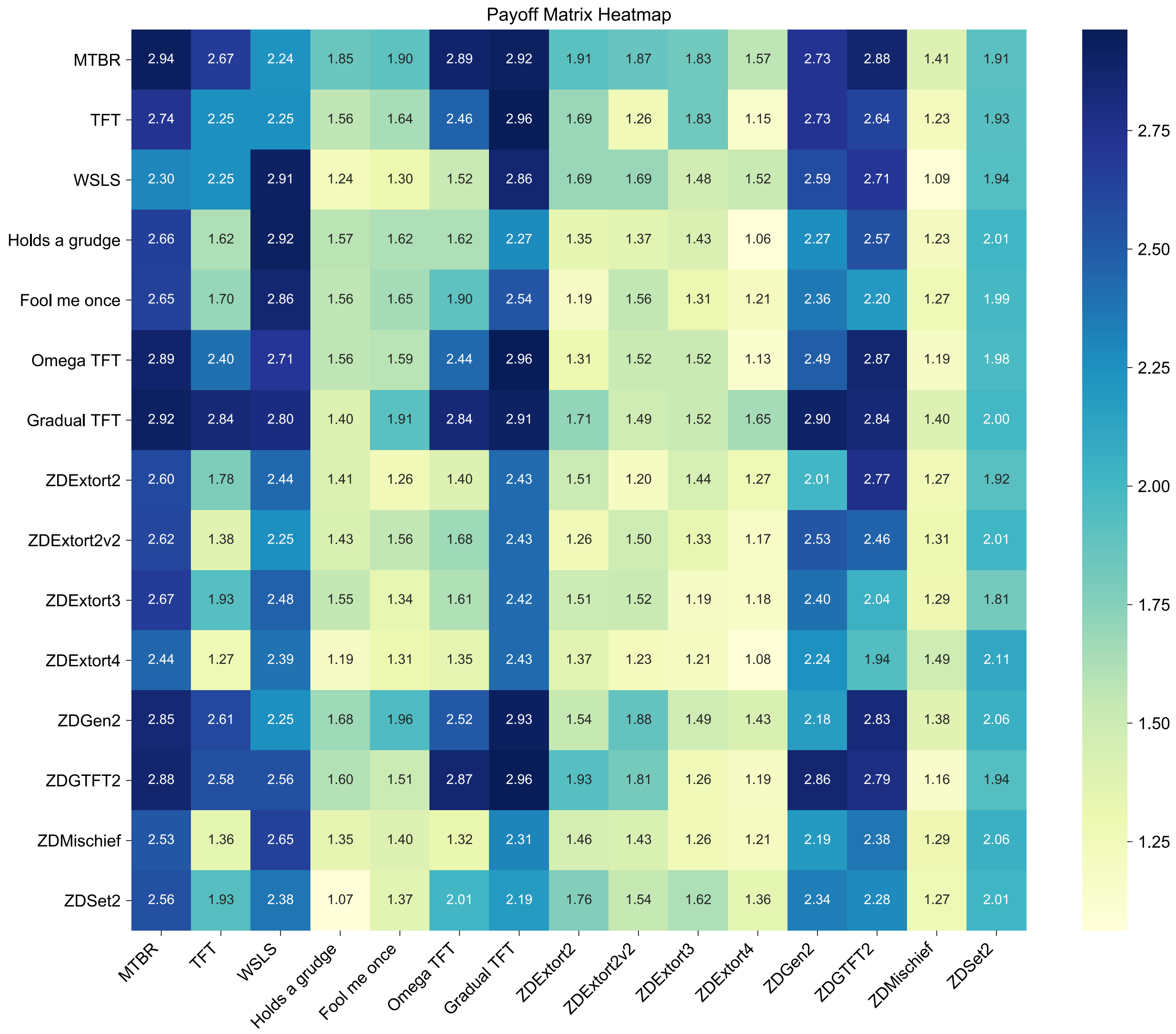

Supplementary Figure 2: Payoff matrix for strategy interactions in the iterated Prisoner's Dilemma. The heatmap shows the average payoffs obtained by 15 different strategies (listed on the x and y axes) when interacting with each other over 20 rounds of the iterated Prisoner's Dilemma. Each cell represents the average payoff of the row strategy when interacting with the column strategy. The color intensity represents the magnitude of the payoff, with darker colors indicating higher payoffs. The payoff structure is defined by the reward matrix [$R = 3$, $T = 5$, $S = 0$, $P = 1$], where R represents the reward for mutual cooperation, T the temptation payoff for defecting while the other cooperates, S the sucker's payoff for cooperating while the other defects, and P the punishment for mutual defection. Strategies include MTBR, TFT, WSLS, Holds a Grudge, Fool me Once, Omega TFT, Gradual TFT, ZDExtort2, ZDExtort2v2, ZDExtort3, ZDExtort4, ZDGen2, ZDGTFT2, ZDMischief, and ZDSet2. MTBR, our proposed strategy, is represented in the first row and column, allowing for direct comparison with other well-established strategies. The heatmap reveals patterns of strategy performance, showcasing how certain strategies, particularly MTBR, can consistently achieve higher payoffs across various interactions, while others may be more vulnerable to exploitation or perform well only against specific opponents.

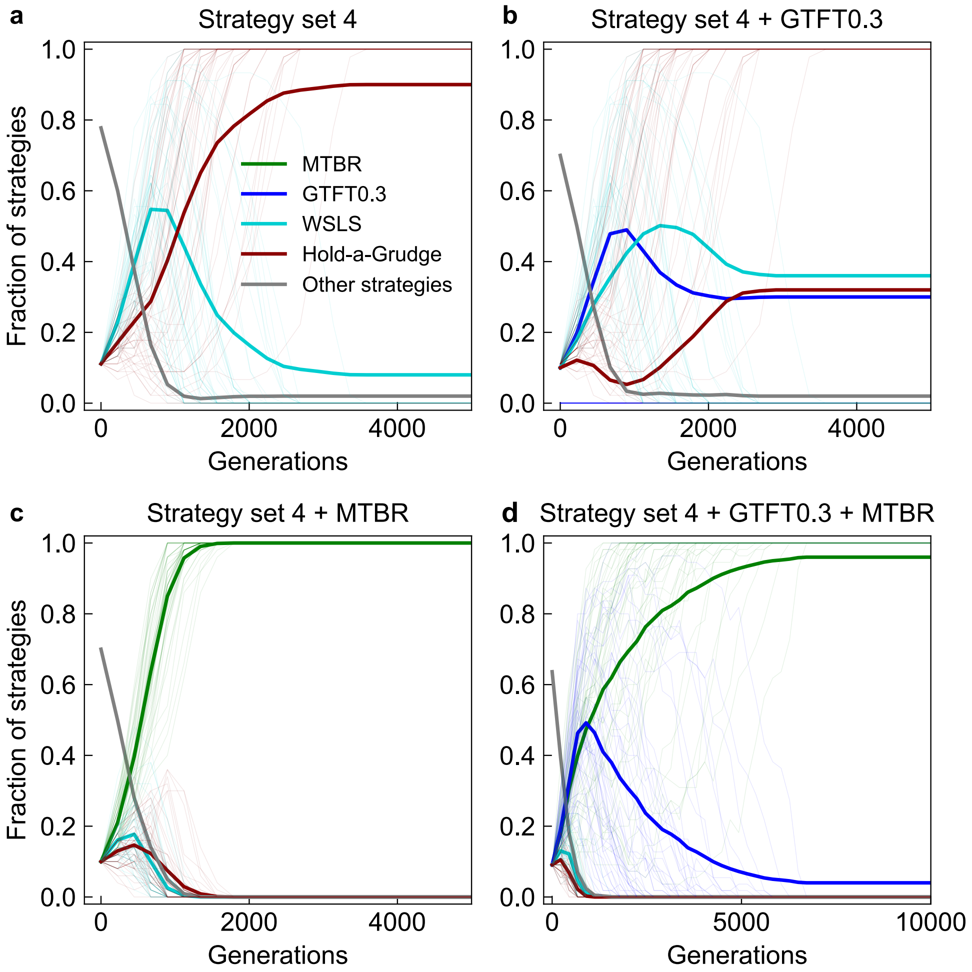

Supplementary Figure 3: MTBR promotes high cooperation in a small and competitive strategy set. We define *Strategy Set 4* consisting of nine strategies: TFT, WSLS, Hold-a-Grudge, ZDExtort2, ZDExtort2v2, ZDExtort3, ZDExtort4, ZDMischief, and AllD. Each strategy is initialized with 5 individuals. Given the relatively small population sizes (ranging from 45 to 55), the evolutionary dynamics tend to converge to monomorphic populations, where a single strategy ultimately dominates. Therefore, each trajectory represents the probability of a strategy fully taking over the population, averaged over 50 independent runs. **a**, Evolutionary outcome under Strategy Set 4 alone. Hold-a-Grudge dominates with 90% probability, followed by WSLS (8%) and AllD (2%). The average payoff at equilibrium is 1.78. **b**, Evolution with Strategy Set 4 plus GTFT0.3. The population stabilizes under GTFT0.3 (30%), WSLS (36%), Hold-a-Grudge (32%), and AllD (2%). The average population payoff increases to 2.44. **c**, Evolution with Strategy Set 4 plus MTBR. MTBR completely takes over the population in all runs (100% dominance), yielding an average payoff of 2.94. **d**, Evolution with Strategy Set 4 plus both GTFT0.3 and MTBR. MTBR dominates in 96% of runs, and GTFT0.3 in 4%. The average population payoff remains high at 2.93. These results show that while GTFT0.3 can moderately promote cooperation in this difficult setting, MTBR consistently achieves near-optimal outcomes by fully stabilizing cooperation, even when coexisting with other cooperative strategies. This underscores MTBR's robustness and superior cooperation-promoting capability under constrained evolutionary conditions.

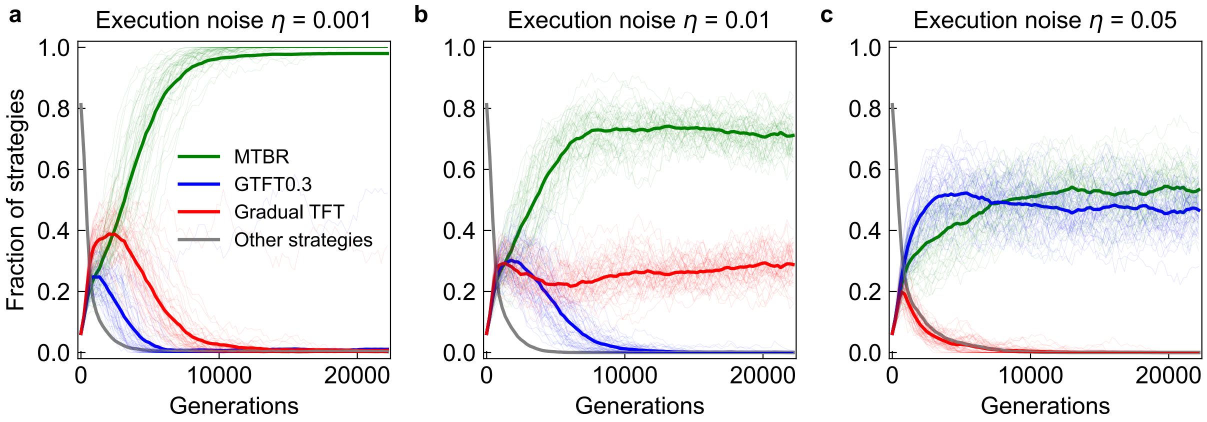

Supplementary Figure 4: MTBR remains dominant under behavioral execution errors. We examine the evolutionary dynamics when agents occasionally mis-execute their intended actions with probability η, introducing behavioral execution noise. Three levels of noise are considered: $\eta = 0.001$, 0.01, and 0.05. **a**, At low noise ($\eta = 0.001$), MTBR remains fully dominant, ultimately taking over the entire population. **b**, When $\eta = 0.01$, MTBR stabilizes at approximately 72%, coexisting with GradualTFT. **c**, When $\eta = 0.05$, GradualTFT rapidly disappears, and MTBR and GTFT0.3 become the prevailing strategies, stabilizing around 53% and 47% respectively. Each trajectory represents an average over 50 independent simulation runs. All other parameter settings are consistent with those used in Fig. 3 of the main text.

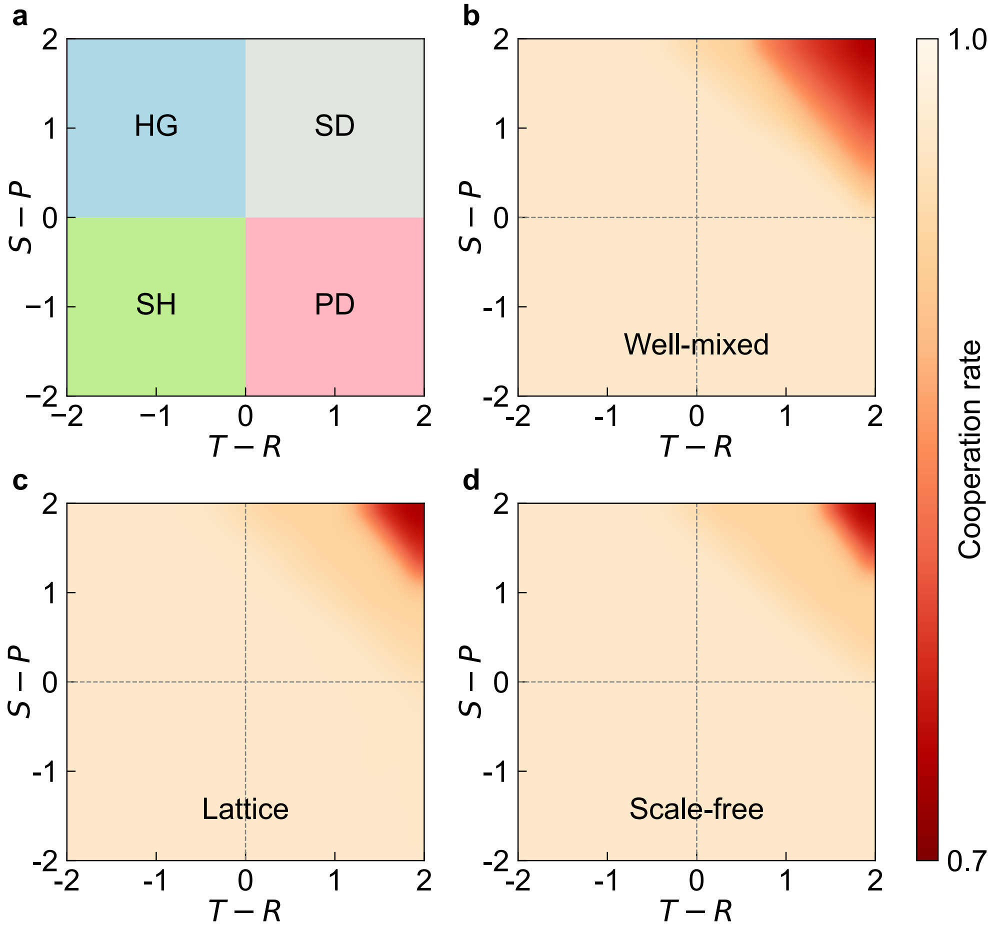

Supplementary Figure 5: Population cooperation rate in evolutionarily stable states under various network structures and payoff matrices. **a**, The positioning of four different game types in parameter space, where Greedy ($T - R$) is on the x-axis and Unfearful ($S - P$) is on the y-axis. **b**, **c**, **d**, Each colored block illustrates the population cooperation rate in evolutionarily stable states within a well-mixed population, lattice grid network, and scale-free network, respectively, under different payoff matrices ($R = 3, P = 1$). The color gradient, shifting from yellow to green to dark blue, represents the decline of population cooperation rate from 1.0 to 0.7 in evolutionarily stable states, with T on the x-axis and S on the y-axis. In the study of games under complex network structures, understanding the population cooperation rate is crucial. We observe that near the line $2R = T + S$, the population cooperation rate remains close to 1. Lower population cooperation rates occur in regions where the values of S and T are both higher. Our simulation verification shows that near the line $2R = T + S$, the population exhibits a mixed state of GTFT0.3 and GradualTFT. However, in the region further to the upper right where $2R < T + S$, the population becomes a mixture of MTBR and TFT. Due to the strong cooperative tendency of GTFT0.3 and GradualTFT, the population cooperation rate also approaches 1 near the line $2R = T + S$. In the region where $2R < T + S$, as the proportion of TFT in the population increases, the population cooperation rate gradually decreases to around 0.7. All data points represent the average of over 100 repeated experiments. Parameters: $\delta = 1, N = 10{,}000$.

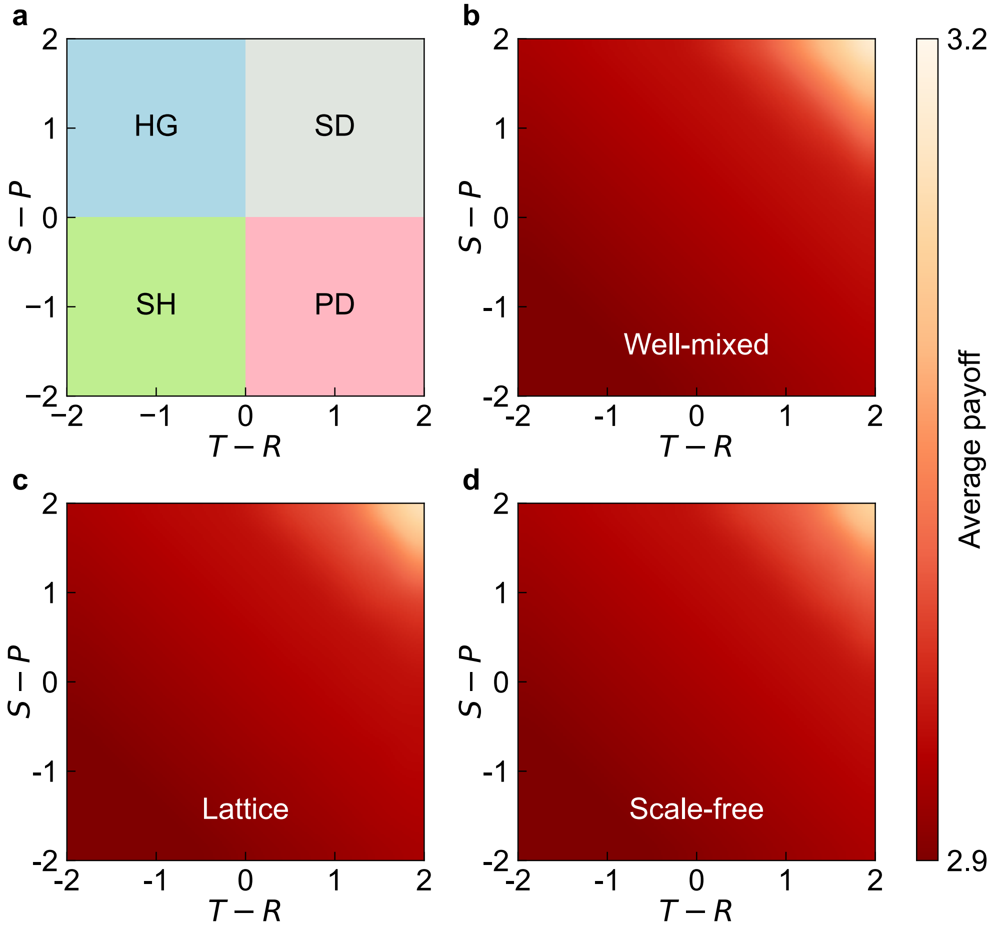

Supplementary Figure 6: Average payoff in evolutionarily stable states under various network structures and payoff matrices. **a**, The positioning of four different game types in parameter space, where Greedy ($T - R$) is on the x-axis and Unfearful ($S - P$) is on the y-axis. **b**, **c**, **d**, Each colored block illustrates the average payoff in evolutionarily stable states within a well-mixed population, lattice grid network, and scale-free network, respectively, under different payoff matrices ($R = 3, P = 1$). The color gradient, shifting from yellow to green to dark blue, represents the decline of average payoff from 3.2 to 2.9 in evolutionarily stable states, with T on the x-axis and S on the y-axis. In addition to population cooperation rate, we also focus on another important metric—the average payoff of all individuals in the population. We found that the trend of average payoff changes is completely opposite to that of the population cooperation rate. The reason is that when $2R < T + S$, both individuals obtaining cooperation and defection behaviors will achieve higher payoffs than mutual cooperation. In this region, the payoff of mutual cooperation is not as good as the stable "cooperate-defect, defect-cooperate" cycle. This also explains the equilibrium reached between MTBR and TFT in this region—when identical individuals meet, TFT gains higher payoff when they randomly choose "cooperate" and "defect" in the first round (see Fig. 1a), and MTBR gains higher payoff when both sides randomly defect in the first round (see Fig. 1b). This interplay explains the strategic variations, highlighting the complexity of interactions and outcomes within these game settings. All data points represent the average of over 100 repeated experiments. Parameters: $\delta = 1, N = 10,000$.

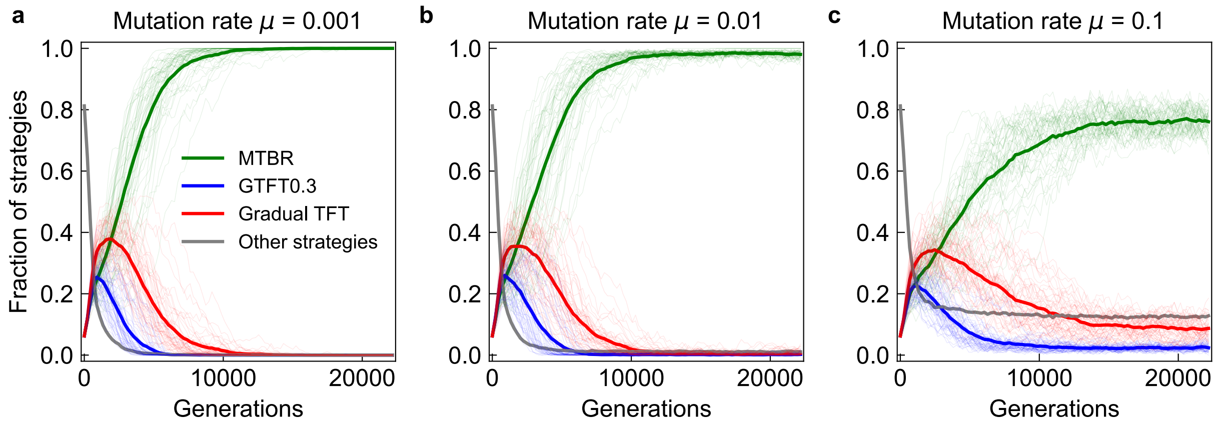

Supplementary Figure 7: Effect of mutation on the evolutionary stability of MTBR. We tested three representative mutation rates: $\mu = 0.001$, 0.01, and 0.1. **a**, When $\mu = 0.001$, the evolutionary dynamics are nearly identical to the no-mutation case, with MTBR rapidly dominating the population. **b**, At $\mu = 0.01$, MTBR remains dominant, though the convergence speed is slightly reduced. **c**, At $\mu = 0.1$, persistent strategic diversity emerges: MTBR occupies 77% of the population, while GradualTFT and GTFT0.3 maintain stable proportions of 9% and 2%, respectively. This pattern aligns with theoretical expectations that strong mutation prevents fixation and stabilizes diverse cooperative equilibria. Each trajectory represents an average over 50 independent simulation runs. All other parameter settings are consistent with those used in Fig. 3 of the main text.

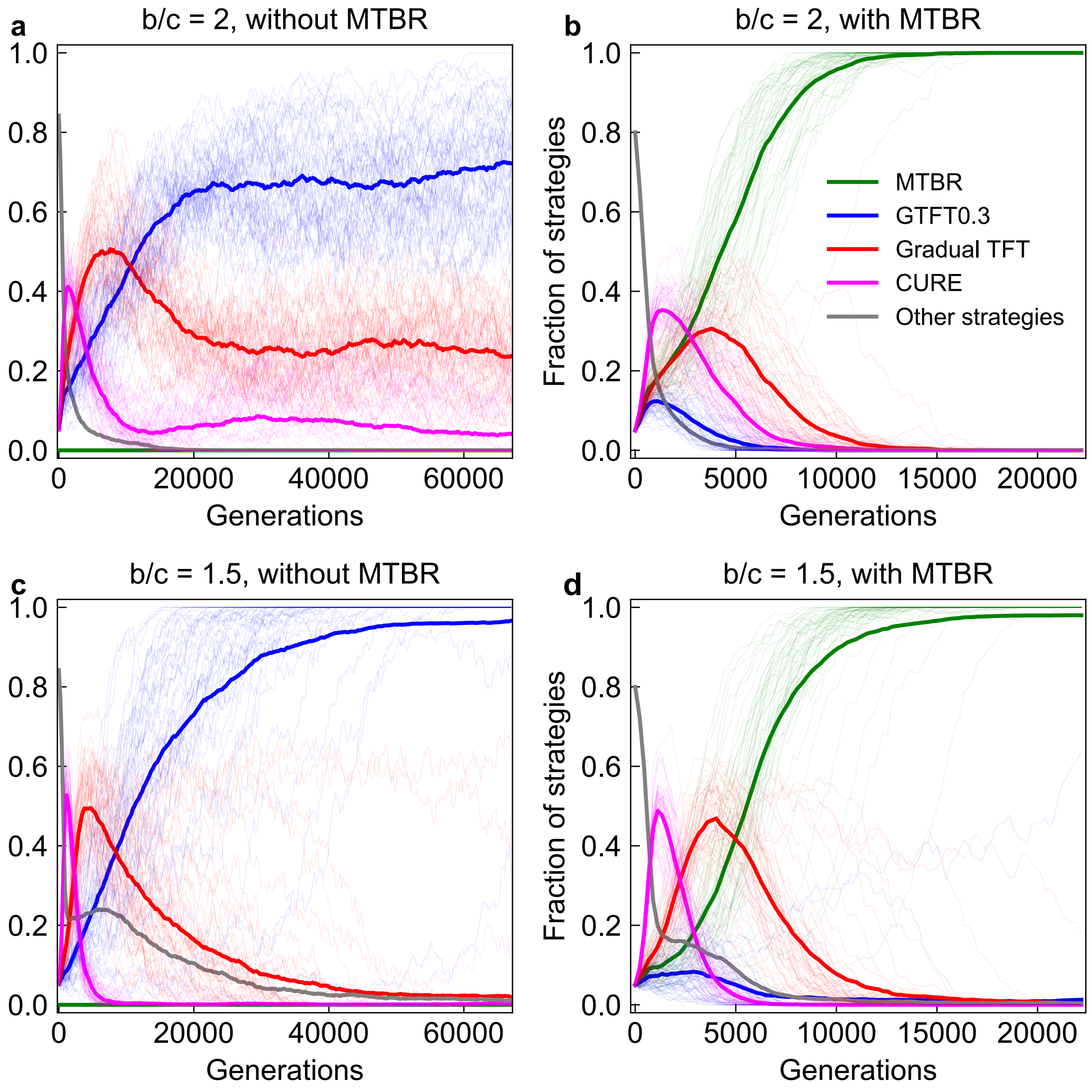

Supplementary Figure 8: MTBR dominates in the presence of advanced longer-memory strategies. We introduce Strategy Set 3, which includes three advanced strategies (namely CURE, AON2, and Reactive-2-Partner) and AllD in addition to the 15 strategies from Strategy Set 2 described in the main text. To evaluate MTBR's performance, we compare two evolving populations: one composed solely of Strategy Set 3 (a for payoff ratio $b/c = 2$, c for $b/c = 1.5$), and the other composed of Strategy Set 3 plus MTBR (b for $b/c = 2$, d for $b/c = 1.5$). Each simulation begins with 33 individuals per strategy. **a**, For $b/c = 2$, the population converges to a mixed equilibrium where GTFT0.3, GradualTFT, and CURE stabilize at 72%, 24%, and 4%, respectively. **b**, When $b/c = 2$ and MTBR is included, it ultimately dominates the entire population. **c**, For $b/c = 1.5$, GTFT0.3 dominates the population, stabilizing at approximately 97%. **d**, With $b/c = 1.5$ and MTBR present, it stabilizes at around 98% of the population. These results demonstrate that MTBR withstands competition from sophisticated, longer-memory strategies. Notably, CURE tends to perform relatively well in the early stages of evolution. Each trajectory represents an average over 50 independent simulation runs. All other parameter settings are consistent with those used in Fig. 3 of the main text.

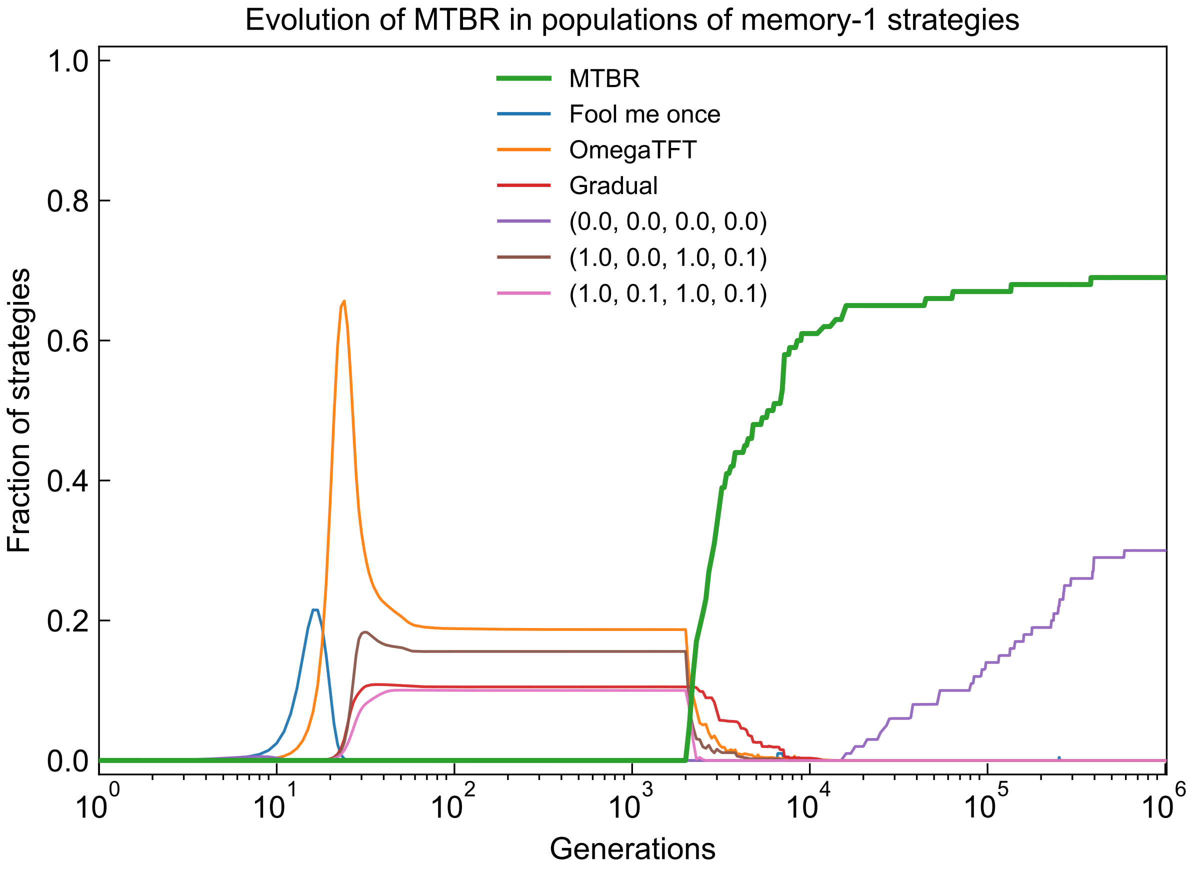

Supplementary Figure 9: Evolutionary dynamics of MTBR within a memory-1 strategy class. We examine an evolutionary system that includes the original eight focal strategies (MTBR, Hold-a-Grudge, Fool-me-Once, OmegaTFT, GradualTFT, CURE, AON2, and Reactive-2), and 11^4 additional memory-one strategies. Initially, all strategies are introduced at equal abundance. In the long run, MTBR becomes the sole surviving strategy in approximately 70% of independent runs, while ALLD dominates in the remaining 30%. For clarity, we present only those strategies that reach a frequency of at least 10% at some point during the evolutionary process. Each curve reports the average frequency over 100 independent runs. All other parameter settings are consistent with those used in Fig. 3 of the main text.

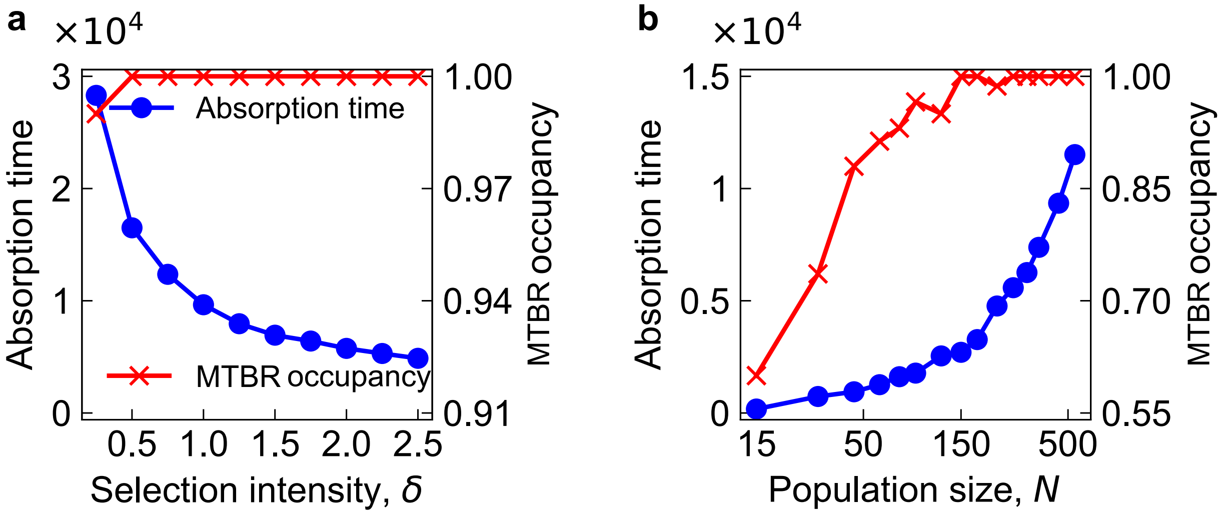

Supplementary Figure 10: Impact of selection intensity and population size on evolutionary dynamics. The blue lines marked with dots represent the absorption time (generations) required for the population to reach evolutionarily stable states. The red lines marked with "x" symbols represent the fraction of the population occupied by MTBR in evolutionarily stable states. **a**, The influence of selection intensity s on the absorption time and the occupancy probability of MTBR in evolutionarily stable states for a fixed population size of $N = 500$. As selection intensity increases, we observe a corresponding decrease in absorption time. Notably, at $N = 500$, MTBR demonstrates the ability to stably occupy the population. **b**, The impact of population size N on the absorption time and the occupancy probability of MTBR in evolutionarily stable states for a fixed selection intensity of $\delta = 1$. Larger population sizes result in slower strategy dissemination and longer absorption times. In smaller populations, exploitative strategies have a competitive advantage. As the population size increases, the high returns resulting from interactions between cooperative strategies gradually emerge. All data points represent the average of over 100 repeated experiments.

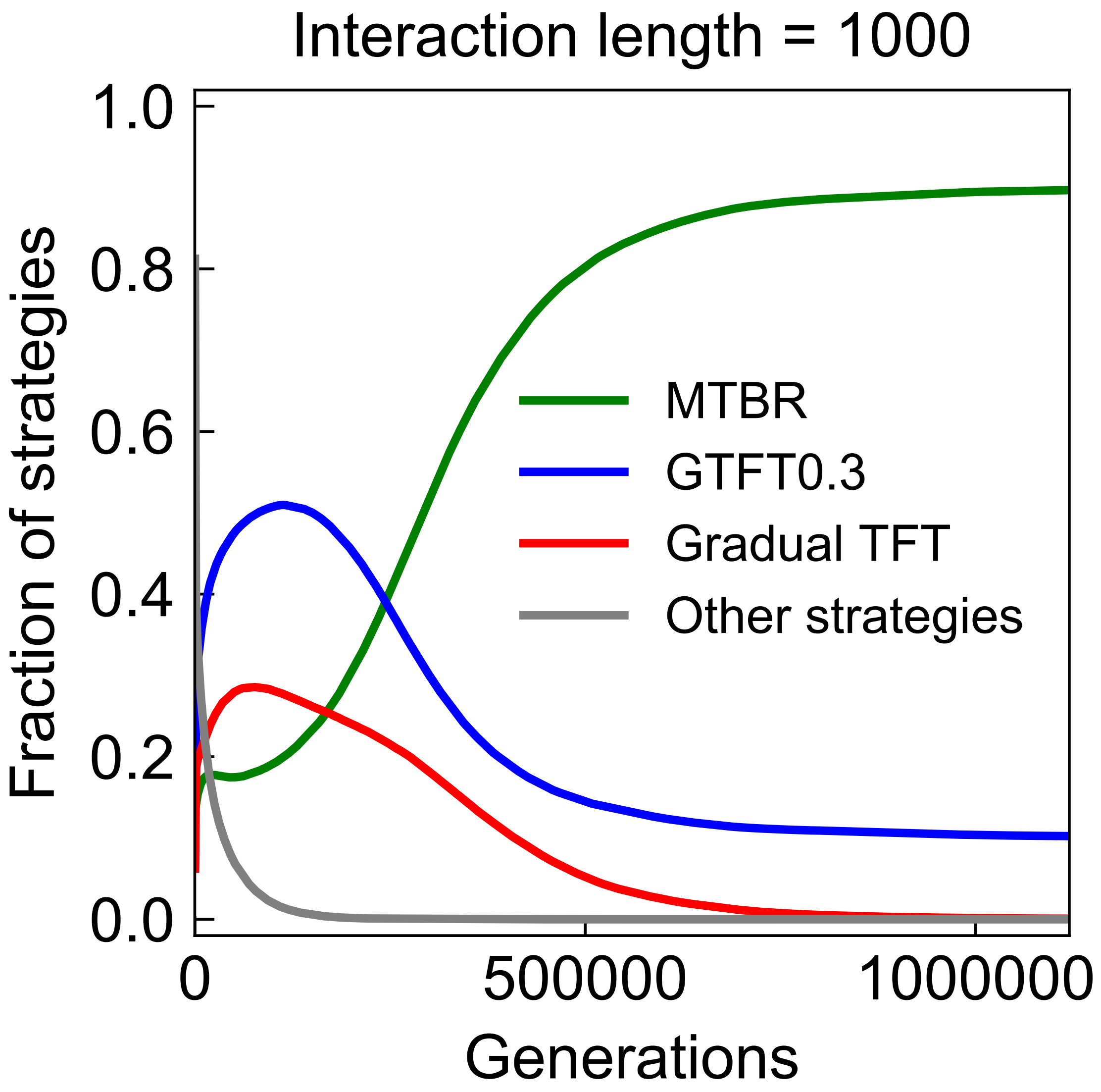

Supplementary Figure 11: Evolutionary dynamics with long interactions. We consider an evolving population based on strategy set 2 from the main text. The interaction length is fixed at 1,000 rounds. Initially, all strategies are equally abundant. Each line shows the average frequency across 1,000 independent runs. All other parameter settings are consistent with those used in Fig. 3 of the main text.
