# Supplementary material for: A multi-agent reinforcement learning framework for exploring dominant strategies in iterated and evolutionary games
Source: Nat Commun. 2025 Dec 8;17:490. doi: 10.1038/s41467-025-67178-6 (PMC12804933; doi:10.1038/s41467-025-67178-6)
Supplement: Supplementary file 2 — Reporting Summary [file 41467_2025_67178_MOESM2_ESM.pdf]

nature portfolio

Corresponding author(s): Long Wang & Qi Su

Last updated by author(s): Nov 24, 2025

Reporting Summary

Nature Portfolio wishes to improve the reproducibility of the work that we publish. This form provides structure for consistency and transparency in reporting. For further information on Nature Portfolio policies, see our Editorial Policies and the Editorial Policy Checklist.

Statistics

For all statistical analyses, confirm that the following items are present in the figure legend, table legend, main text, or Methods section.

n/a	Confirmed	
☐	☒	The exact sample size (*n*) for each experimental group/condition, given as a discrete number and unit of measurement
☐	☒	A statement on whether measurements were taken from distinct samples or whether the same sample was measured repeatedly
☒	☐	The statistical test(s) used AND whether they are one- or two-sided<br>*Only common tests should be described solely by name; describe more complex techniques in the Methods section.*
☒	☐	A description of all covariates tested
☒	☐	A description of any assumptions or corrections, such as tests of normality and adjustment for multiple comparisons
☒	☐	A full description of the statistical parameters including central tendency (e.g. means) or other basic estimates (e.g. regression coefficient) AND variation (e.g. standard deviation) or associated estimates of uncertainty (e.g. confidence intervals)
☒	☐	For null hypothesis testing, the test statistic (e.g. *F*, *t*, *r*) with confidence intervals, effect sizes, degrees of freedom and *P* value noted<br>*Give P values as exact values whenever suitable.*
☒	☐	For Bayesian analysis, information on the choice of priors and Markov chain Monte Carlo settings
☒	☐	For hierarchical and complex designs, identification of the appropriate level for tests and full reporting of outcomes
☒	☐	Estimates of effect sizes (e.g. Cohen's *d*, Pearson's *r*), indicating how they were calculated

Our web collection on statistics for biologists contains articles on many of the points above.

Software and code

Policy information about availability of computer code

Data collection	No empirical data were collected; all results are based on numerical simulations. First, a customized multi-agent reinforcement-learning framework implemented in Python 3.8.5 is used to search over the space of strategies and discover the memory-two bilateral reciprocity (MTBR) strategy. Second, simulation code implemented in Python 3.8.5 and Julia 1.9.4 is used to evaluate fixed strategies—including MTBR and the benchmark strategies—in repeated Prisoner's Dilemma tournaments and, where applicable, evolutionary settings, thereby generating the numerical simulation outputs underlying all main-text and supplementary figures. The full and up-to-date codebase is available in the GitHub repository (https://github.com/YuzukiWang/multiagent_q_learning_for_evolutionary_games). Key Python dependencies are NumPy 1.24.4 and Matplotlib 3.7.5 together with standard-library modules, and key Julia dependencies include ArgParse, DataFrames, Distributions, IterativeSolvers and StatsBase, together with the standard libraries LinearAlgebra, Random, SparseArrays and Printf.
Data analysis	The performance analysis of MTBR and the benchmark strategies, as well as the generation of all figures, is carried out using Python 3.8 and Julia 1.9.4. Analysis and plotting scripts that reproduce all reported results from the stored numerical simulation outputs are included in the GitHub repository (https://github.com/YuzukiWang/multiagent_q_learning_for_evolutionary_games). The main Python dependencies are NumPy 1.24.4 and Matplotlib 3.7.5 together with standard-library modules, and the main Julia dependencies include DataFrames, Distributions, IterativeSolvers and ArgParse, together with the standard libraries LinearAlgebra, Random, SparseArrays, StatsBase and Printf.

For manuscripts utilizing custom algorithms or software that are central to the research but not yet described in published literature, software must be made available to editors and reviewers. We strongly encourage code deposition in a community repository (e.g. GitHub). See the Nature Portfolio guidelines for submitting code & software for further information.

Data

Policy information about availability of data

All manuscripts must include a data availability statement. This statement should provide the following information, where applicable:

- Accession codes, unique identifiers, or web links for publicly available datasets
- A description of any restrictions on data availability
- For clinical datasets or third party data, please ensure that the statement adheres to our policy

All data needed to evaluate the conclusions in the paper are present in the paper and/or the Supplementary Materials. The associated code and supplementary materials are available at: https://github.com/YuzukiWang/multiagent_q_learning_for_evolutionary_games.

Research involving human participants, their data, or biological material

Policy information about studies with human participants or human data. See also policy information about sex, gender (identity/presentation), and sexual orientation and race, ethnicity and racism.

Reporting on sex and gender	This study does not involve human participants or their data; therefore, sex and gender reporting is not applicable.
Reporting on race, ethnicity, or other socially relevant groupings	This study does not involve human participants or their data; therefore, reporting on race, ethnicity, or other socially relevant groupings is not applicable.
Population characteristics	This study does not involve human participants; therefore, population characteristics are not applicable.
Recruitment	This study does not involve human participants; therefore, no recruitment process was conducted.
Ethics oversight	This study does not involve human participants or their data; therefore, ethical oversight is not applicable.

Note that full information on the approval of the study protocol must also be provided in the manuscript.

Field-specific reporting

Please select the one below that is the best fit for your research. If you are not sure, read the appropriate sections before making your selection.

☐ Life sciences ☒ Behavioural & social sciences ☐ Ecological, evolutionary & environmental sciences

For a reference copy of the document with all sections, see nature.com/documents/nr-reporting-summary-flat.pdf

Behavioural & social sciences study design

All studies must disclose on these points even when the disclosure is negative.

Study description	This study utilizes a multi-agent reinforcement learning approach to explore dominant strategies in iterated and evolutionary games. The focus is on exploring strategies that maximize cooperation and social welfare(average payoffs) in evolving populations of agents engaged in repeated interactions. This is a study quantitative simulated data. No human participants or qualitative methods are involved.
Research sample	Not applicable to human subjects. The study uses simulated agents and predefined mentor strategies as specified in the manuscript (e.g., a population of learning agents interacting with mentors in repeated Prisoner's Dilemma games under fixed payoff parameters). These settings provide a controlled and reproducible environment to evaluate strategy performance.
Sampling strategy	Agents were randomly initialized with a variety of strategies from a predefined strategy set. The strategies were chosen based on previous studies in evolutionary game theory as well as strategies discovered during the experiment. In each simulation episode, one learning agent and one opponent (agent or mentor) are selected at random from their respective pools. In the first round of each game, the initial action is randomized between cooperation and defection to differentiate strategies in the noise-free setting. Independent runs use different random seeds.
Data collection	No empirical data were collected. Agent states, actions, and payoffs are logged programmatically during simulations implemented in Python 3.8.5 on Windows 11 (core dependencies: NumPy 1.24.4, Matplotlib 3.7.5, plus Python standard-library modules). No physical instruments or observers were involved; blinding is not applicable. Data was collected from each agent's actions (cooperate or defect) and their accumulated payoffs over multiple rounds. The performance of strategies was tracked over time to observe their evolution and dominance in the population.
Timing	Simulations were executed programmatically; calendar start/stop dates are not applicable. To reach an evolutionarily stable state, each simulation in the conclusion section typically involves tens of thousands of interactions (temporal scale). The data presented in the conclusion section represent the averages of 50 or 100 repeated experiments (simulations). In these simulations, agents interact based on fixed topologies (well-mixed populations, lattice, or scale-free networks), ensuring diverse spatial interaction patterns.
Data exclusions	No data exclusions were made. All simulation runs were included in the analysis.

Non-participation	In this study, there were no participants involved, as the research is conducted entirely through agent-based simulations. Therefore, no participants were dropped out, and the response rate is not applicable.
Randomization	The initialization of agent strategies and network topology was randomized to test the robustness of the results across different conditions. Randomization was applied to opponent matching (uniform random selection from agent/mentor pools), to the initial action in the first round (cooperate or defect), and to random seeds across independent runs.

Reporting for specific materials, systems and methods

We require information from authors about some types of materials, experimental systems and methods used in many studies. Here, indicate whether each material, system or method listed is relevant to your study. If you are not sure if a list item applies to your research, read the appropriate section before selecting a response.

Materials & experimental systems

n/a	Involved in the study
☒	☐ Antibodies
☒	☐ Eukaryotic cell lines
☒	☐ Palaeontology and archaeology
☒	☐ Animals and other organisms
☒	☐ Clinical data
☒	☐ Dual use research of concern
☒	☐ Plants

Methods

n/a	Involved in the study
☒	☐ ChIP-seq
☒	☐ Flow cytometry
☒	☐ MRI-based neuroimaging

Plants

Seed stocks	This study does not involve the use of seed stocks.
Novel plant genotypes	This study does not involve the use or development of novel plant genotypes.
Authentication	This study does not involve plant material requiring authentication.
